# 420. Immunization Impact on Serotype Distribution of Invasive Pneumococcal Disease - Playing the Serotype Whack-A-Mole!

**DOI:** 10.1093/ofid/ofae631.134

**Published:** 2025-01-29

**Authors:** Delma J Nieves, Stephanie Osborne, Michele Cheung, Matt Zahn, Antonio C Arrieta

**Affiliations:** CHOC Children's Hospital, Orange, CA; Children's Hospital of Orange County, Orange, California; Orange County Health Care Agency, Santa Ana, CA; Orange County Health Care Agency, Santa Ana, CA; Children's Hospital of Orange County, Orange, California

## Abstract

**Background:**

Implementation of 7-valent pneumococcal vaccine (PCV7) resulted in significant reduction of invasive pneumococcal disease (IPD) followed by a subsequent increase in IPD by non-vaccine (n-Vac) serotypes (ser) (19A, 7F, 3) with increased severity and antimicrobial resistance. Approved following immunogenicity data, 13-valent PCV (PCV13) adoption reduced IPD again. Surveillance was strongly recommended by the CDC. We present 13 years of countywide (Orange County, CA) surveillance of IPD and serotypes.

Serotype distribution of IPD by Year

**Figure:**
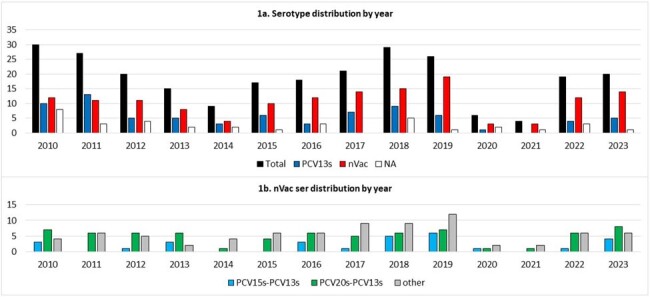

**Methods:**

Prospective (1/2010 - 12/2023) countywide study of patients < 18 years old with culture proven IPD. Demographic, clinical course and immunization data were collected. Serotyping was by Quelung method. Antibiotic susceptibilities were obtained.

**Figure:**
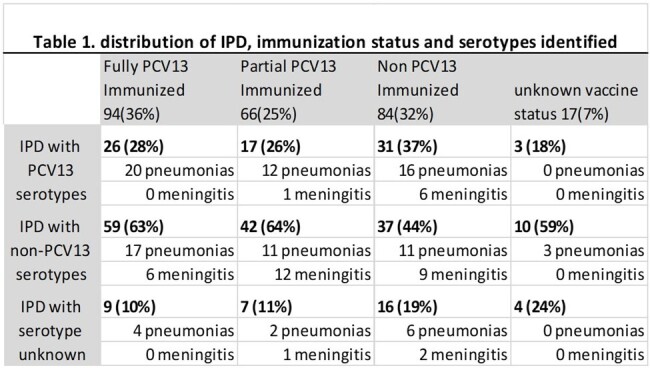

**Results:**

During the study period 261 patients were identified; 42% female, median age 3.7 years, 52% < 5 years old. IPD rate per 10,000 decreased by 70% (0.408 – > 0.123) from 2010 - 2014 and increased steadily from 2015 to 2019 (peaking at 0.403). As COVID19 pandemic social distancing measures were adopted, IPD rate decreased by 86% (0.057 in 2021); it has since rapidly increased again (0.285) after lifting social distancing measures. Ser was known for 86%; 15B and 22F were the most common nVac ser seen (8% and10% respectively). Ser distributions by year are seen in Figure 1. Table 1 shows the distribution of IPD with immunization status and where the pneumonias and meningitis occurred. Pneumonia (102 [39%]) remains the most common IPD (30% with empyema); we saw a rise of meningitis cases after 2018, 1 was ser3, 13 were n-Vac ser and 3 unknown. IPD attributed mortality occurred in 8 (3%) patients; 4 were due to PCV13 ser. Penicillin resistance (meningitis) increased from 21% to 31%; penicillin non-susceptible (MIC >1) remained unchanged. New 15 and 20 valent ser include 12% and 19% of emerging n-Vac serotypes.

**Conclusion:**

Similar to PCV-7, PCV-13 implementation resulted in an initial significant decrease in IPD, mainly due to 5 of the 6 additional ser included in PCV-13, ser 3 was not significantly affected. This was followed by resurgence of IPD, mainly to n-VAC serotypes. Of our IPD since 2010, 65% are from ser now included in 20-valent PCV. IPD attributable mortality remains high. Active surveillance following higher valent PCVs remains important.

**Disclosures:**

**All Authors**: No reported disclosures

